# Early invasive vulvar squamous cell carcinoma arising in a woman with vulvar pemphigus vulgaris and systemic lupus erythematosus

**DOI:** 10.1186/1471-2407-10-324

**Published:** 2010-06-23

**Authors:** Giuseppe Bifulco, Vincenzo D Mandato, Roberto Piccoli, Pierluigi Giampaolino, Chiara Mignogna, Michele D Mignogna, Luigi Costagliola, Carmine Nappi

**Affiliations:** 1 Department of Gynecology and Obstetrics, and Pathophysiology of Human Reproduction, University of Naples "Federico II", Italy; 2 Department of Biomorphological and Functional Sciences, Pathology Section, University of Naples "Federico II", Italy; 3 Oral Medicine Unit, Department of Odontostomatologica and Maxillo-facial Science, University of Naples "Federico II", Italy

## Abstract

**Background:**

Pemphigus vulgaris (PV) is an autoimmune blistering disease of the skin and mucous membranes. Genital involvement occurs when most other common sites are concurrently affected or are in remission. Systemic lupus erythematosus (SLE) is an autoimmune disease that may affect many parts of the body and the skin with occasional bullous lesions. Pemphigus vulgaris and SLE may be associated, albeit rarely. Here, we report the first case of a woman affected with SLE presenting with early invasive squamous cell carcinoma (SCC) arising from Pemphigus Vulgaris of the vulva.

**Case presentation:**

A 27-year-old Caucasian woman was admitted to our Gynaecology Unit for bleeding vegetant lesions of the vulva. Her history was characterized by systemic lupus erythematosus and PV. Biopsy showed concomitant PV and vulvar intraepithelial neoplasia (VIN) grade 3. One month later a new biopsy revealed progression from VIN 3 to early SCC. Despite chemotherapy, no remission of disease was observed. She died six months after diagnosis

**Conclusion:**

Our case underlines PV as another chronic inflammatory disease of the lower genital tract predisposing to VIN-SCC. It suggests the need for careful follow-up of patients with chronic inflammatory disease, especially when concomitant autoimmune disorders are present. Moreover, a biopsy should be always performed if there are PV lesions because of the possibility of neoplastic disease.

## Background

Pemphigus vulgaris (PV) is an autoimmune blistering disease of the skin and mucous membranes characterized by the presence of autoantibodies targeting desmoglein 3, a surface antigen of keratinocytes involved in maintaining cell-cell junctions [1]. Its incidence is 0.1-3.2 cases per 100,000 individuals per year. PV typically runs a chronic course, with blisters, painful erosions and ulcers on the mucosa and skin [2,3]. The sites most commonly involved are the oral mucosa, pharynx, larynx, oesophagus, conjunctiva and anal mucosa [1-4]. Involvement of the genital tract in women with PV has rarely been reported [5]. Usually, genital involvement occurs when most other common sites are concurrently affected or are in remission [1,6].

Systemic lupus erythematosus (SLE) is an autoimmune disease that may affect many parts of the body and the skin with occasional bullous lesions. Pemphigus vulgaris may exceptionally be associated with other blistering diseases such as SLE [7].

Here, we report a rare case of a woman affected with SLE presenting with early invasive squamous cell carcinoma (SCC) arising from Pemphigus Vulgaris of the vulva.

## Case presentation

A 27-year-old Caucasian woman was admitted to our Gynaecology Unit (February 2007) for bleeding vegetant lesions of the vulva. She had no family history of autoimmune disease. Her history was characterized by concomitant SLE and recurrent blisters and erosions affecting the oral mucosa and skin diagnosed as severe PV. She had been treated with high and prolonged doses of both systemic corticosteroids and other immunosuppressive drugs (Prednisone 100 mg/day × 69 months; Azathioprine 80 mg/day × 51 months; Cyclophosphamide 83 mg/day × 15 months; Cyclosporine 300 mg/day × 2 months).

**Figure 1:**
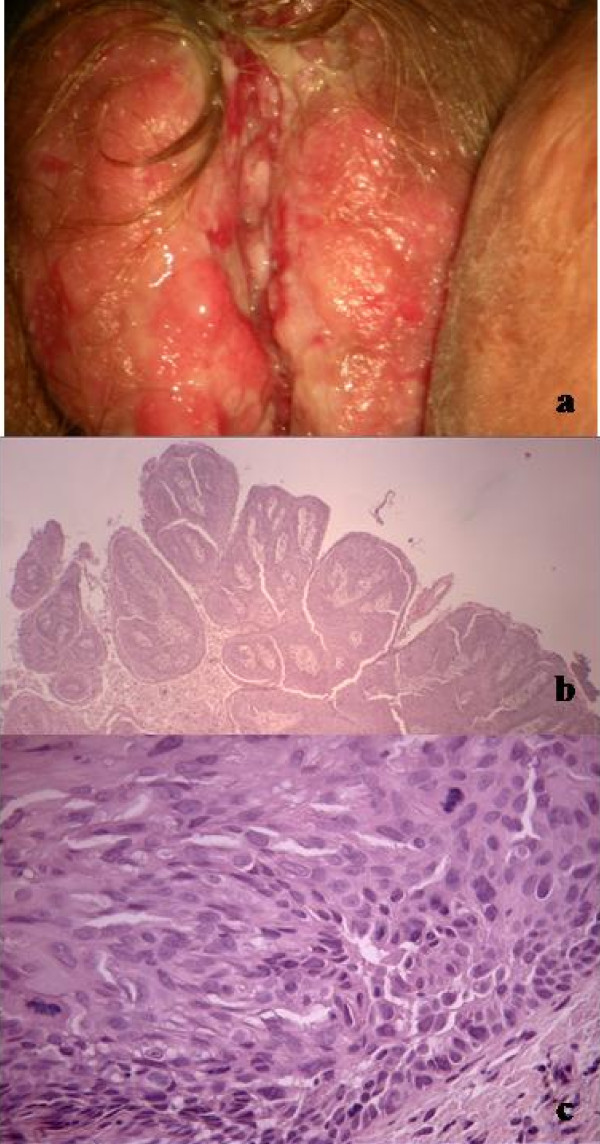
**Macroscopic and microscopy findings at diagnosis**: (a): vulvar bleeding vegetants lesions; (b): micropapillary pattern; (c): acantholysis and mitosis.

She had had necrosis of the femoral and humerus necks, osteortrosis, arthritis, osteoporosis and recurrent infections. Most recently, at gynaecological examination a thick area associated with ulcerative necrotic areas, and atypical vessels were revealed. Vulvar biopsy diagnosed PV. The epidermidis was arranged in a micropapillary pattern with combined aspects of acantholytic and atypical cells. Multiple areas of acantholytic clefts separated the upper part of the epidermidis from the basal keratinocytes. Unusually, dysplastic cells with atypical mitoses were present (Figure 1). Because she was virgin , She never had a pap smear and we did not perform it.

**Figure 2:**
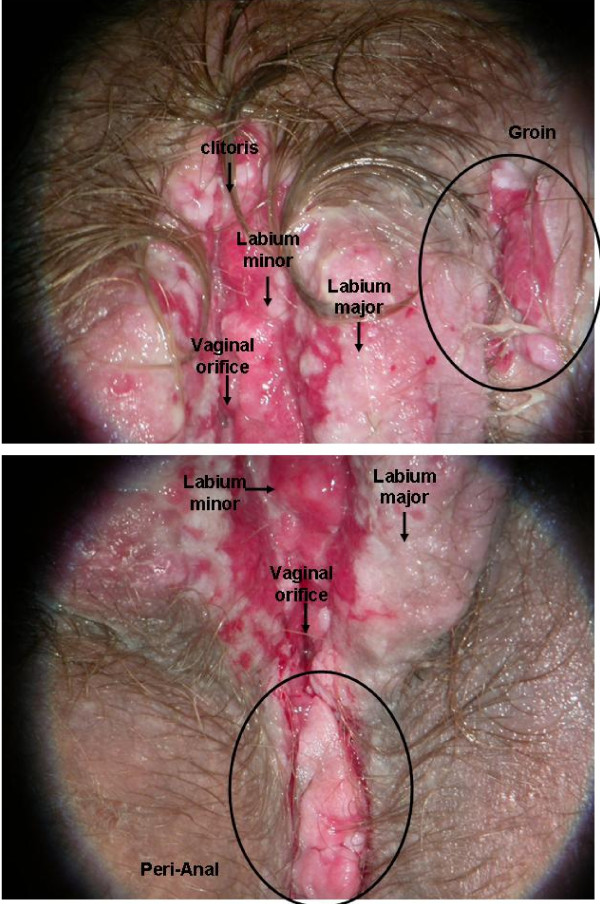
**Macroscopic findings at one month from diagnosis**: lesion was extended to peri-anal area and groin area.

On March 2007 the lesion had extended to the perineal and groin area (Figure 2). Biopsy revealed a vulvar intraepithelial neoplasia (VIN) grade 3 with microinvasive disease. The SCC vertical invasion of the stroma was less than 1 mm.

**Figure 3:**
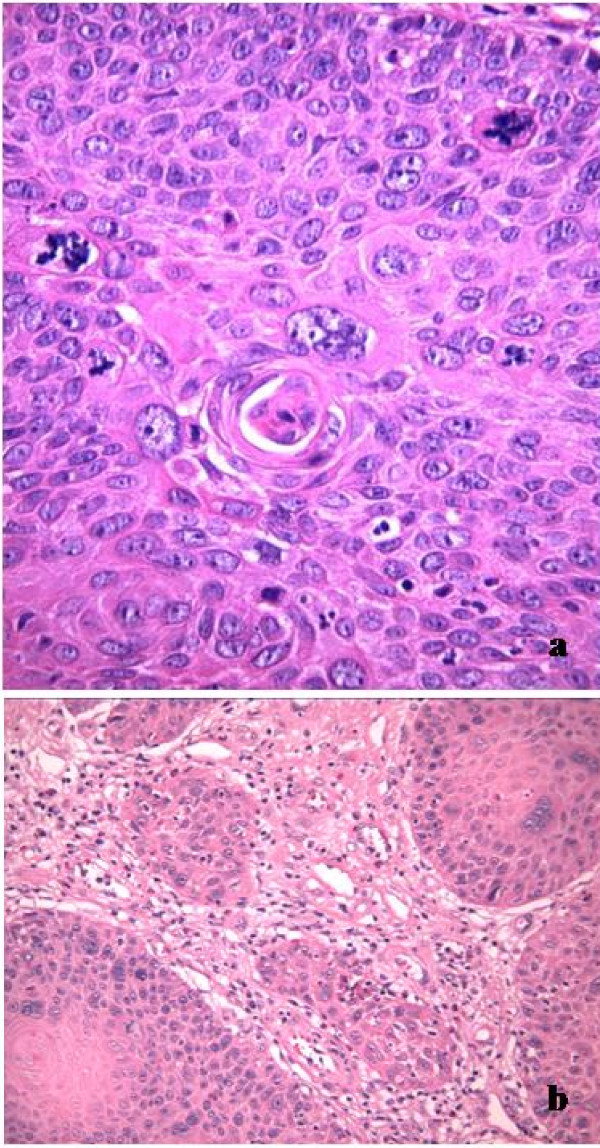
**Microscopy findings at one month from diagnosis**: (a): atypical mitosis; (b): infiltrative aspects were present.

Hyperplastic epidermidis was characterized by the formation of acantholytic clefts and the presence of displastic cells and mitotic activity. Focal infiltrative aspects were present (Figure 3).

Positron Emission Tomography (PET) and Magnetic Resonance Imaging (MRI) showed no metastatic disease.

Current therapy was based on High dose human immunoglobulins (IVIg).

IVIg 5% solution were infused intravenously with an electronic pumping device at a total dose of 2 g/kg per cycle divided into three equal doses, administered over 3 consecutive days. The infusion was administered slowly at not more than 50 mg/kg per hour.

The therapy improved the immunological PV but not the clinical PV. The antibody titer showed a progressive decrease (pre-IVIg titer was of 1:1280; during IVIg titer was of 1: 640; post IVIg titer was of 1:80)

Based on biopsy findings, a wide and deep excision of the primary tumor was required but our patient was classified as ASA IV at preoperative evaluation. She presented a 25 kg weight loss, a worsening of the clinical conditions with a high risk of nonhealing wound and postopearative infection so combined chemotherapy was preferred to surgical approach. She received six cycles of cisplatin plus 5-fluorouracil. The doses and schedule was cisplatin 75 mg/m2 on day 1 and 5-fluorouracil 800 mg/m2 on day 2 to 5 every three weeks.

**Figure 4:**
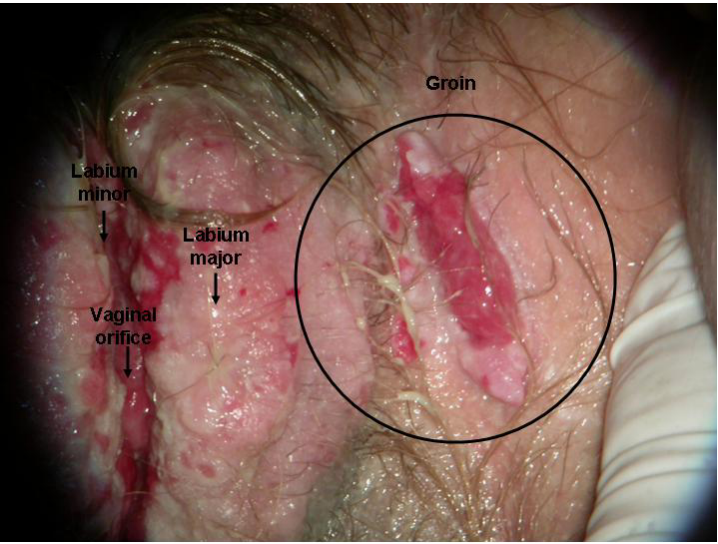
**Macroscopic findings after chemotherapy**: persistent lesions.

**Figure 5:**
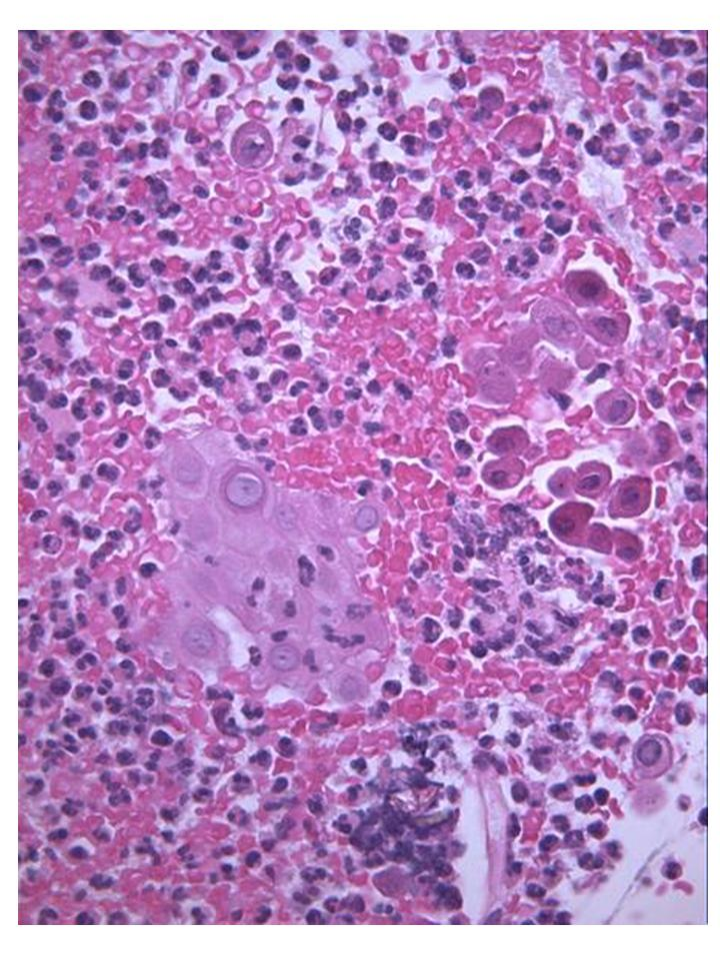
**Microscopy findings after chemotherapy**: atypical and acantolytic cells dispersed into a neutrophils and red blood cells background.

Despite the chemotherapy there was no remission of the lesion, though no local extension was observed (Figure 4). A new biopsy (September 2007) revealed atypical and acantholytic cells dispersed into a neutrophil and red blood cell background (Figure 5). Two months later she died from cardiac arrest due to worsening of her general condition. but no post mortem examination was performed because her parents refused.

## Discussion

Pemphigus vulgaris (PV) is a chronic autoimmune disease with a mortality rate less than 10% following treatment with systemic corticosteroids and immunosuppressive agents [8]. It mainly affects elderly persons. PV is diagnosed on the basis of clinical appearance, histology, and immunofluorescence studies. Histology demonstrates loss of cell-cell adhesion (acantholysis) above the basal layer of the epidermis. Immunoglobulin G is detected on keratinocyte cell surfaces by direct immunofluorescence in nearly all patients, although sera from PV patients contain antibodies that bind to keratinocyte cell surface antigens on indirect immunofluorescence. The pathogenic antibodies are directed against the keratinocyte cell surface molecules desmoglein 1 and 3. An immunoblot assay or enzyme-linked immunosorbent assay may also be used to detect antibodies.

PV is rarely associated with SLE; the association has been reported in the literature in only 4 females [9-12] and 1 male [7]. This is the fifth case of PV associated with SLE. More common blistering skin lesions associated with SLE are paraneoplastic pemphigus, pemphigus erythematosus and drug-induced pemphigus. Paraneoplastic pemphigus consists of painful mucosal ulcerations and a polymorphic blistering eruption on the trunk and extremities, and a characteristic autoantibody pattern that usually resolves after tumour removal [13]. Pemphigus erythematosus is characterized by facial eruption and bullous lesions on the chest, upper back and intertriginous areas with minimal SLE manifestations [14]. Drug-induced pemphigus has a course similar to PV but resolves after discontinuation of the associated drug and is histologically different. PV produces deeper vesicles as a result of the acantholysis in the suprabasal layer of the epidermis, whilst acantholysis in drug-induced pemphigus occurs in the corneal layer.

Therefore, we excluded pemphigus erythematosus because of the severe SLE manifestations in our patient; on the other hand, we excluded drug-induced pemphigus because histologically the epidermis was deeply affected, and because the patient had been treated for several years with corticosteroids without similar signs prior to the diagnosis of PV; finally because she showed immunological improvement after increasing the corticosteroid dose. So we diagnosed our patient as affected by PV. As in three of the previously reported cases of PV associated with SLE [7,11,12], SLE presented before PV in our patient. However, in contrast to the other cases [7], PV persisted in our patient after the worsening of the SLE.

The most unusual feature of our patient was the rapid development of VIN 3 and early invasive squamous cell carcinoma (SCC) arising from PV of the vulva. Two different aetiologies of vulvar cancer are known. One type is mainly seen in younger patients (mean age 55 years), is related to HPV infection and smoking, and is commonly associated with basaloid or warty VIN. In contrast, the more common type is seen mainly in elderly patients (mean age 77 years), is unrelated to smoking or HPV infection, and is seldom associated with concurrent VIN, but there is a high incidence of dystrophic lesions (lichen sclerosus, epithelial hyperplasia, lichen planus) [15-21]. Age is the most important predisposing factor in progression from pre-existing VIN to SCC. The best treatment of VIN is conservative surgical excision and follow-up. In case of vertical invasion of the stroma less than 1 mm, a wide and deep excision of the primary tumor is required [22]. Female genital tract involvement in PV has been limited to a small number of case reports, and even rarer cases of female genital tract PV are associated with squamous cell carcinoma [23].

To our knowledge, no case of SCC arising from PV of the vulva has been reported in the literature.

Despite her young age, our patient presented a rapid progression of VIN to microinvasive disease.

In contrast to other cases reported in the literature, our patient did not present HPV infection. The pathogenic mechanism of VIN and its rapid progression to early SCC may be related to the autoimmune disease, to the use of systemic corticosteroids and immunosuppressive agents for therapy and the consequent deregulation of the immune system.

Autoimmune diseases are triggers of chronic inflammation that is a well known risk factor of developing various type of cancer. Since 1863 Virchow noted a connection between inflammation and cancer. He suggested that the "lymphoreticular infiltrate" reflected the origin of cancer at sites of chronic inflammation. The hallmark of cancer related inflammation include the presence of inflammatory cells and inflammatory mediators in tumor tissues, tissue remodelling and angiogenesis similar to that seen in chronic inflammatory responses and tissue repair [24]. There is strong clinical evidence for an association of chronic inflammation with SCC [25]. SCC can arise from a malignant transformation occurred within a chronic inflammatory focus of ulcerative and non healing wounds [26,27]. This association has been described for LES, epidermolysis bullosa, lichen planus, leg ulcerations [28-34]. Even areas of healed wounds are more susceptible to development SCC. Moreover, inflammation is not only associated with de novo development of SCC but also play a role in his progression [25]. Probably in our patients both LES and PV played a pivotal role in the SCC development.

## Conclusion

Our case underlines PV as another chronic inflammatory disease of the lower genital tract predisposing to VIN-SCC. It suggests the need for careful follow-up of patients with chronic inflammatory disease, especially when concomitant autoimmune disorders are present. Because of the possibility that neoplasms will develop, a biopsy should be always performed in order to prompt diagnosis and the best treatment.

## Abbreviations

PV: Pemphigus vulgaris; SLE: Systemic lupus erythematosus; SCC: Squamous cell carcinoma; VIN: Vulvar intraepithelial neoplasia; PET: Positron Emmision Tomography; MRI: Magnetic Resonance Imaging; IVIg: Human Immunoglobulins.

## Competing interests

The authors declare that they have no competing interests.

## Authors' contributions

GB revised the manuscript and performed follow-up. VDM conceived of the case report, reviewed the literature and wrote the manuscript. RP diagnosed the disease, performed the follow-up, conceived of the case report and revised the manuscript. PG reviewed the literature, obtained the pictures and collected data. CM performed the histological analysis and wrote the manuscript. MM performed the follow-up and revised the manuscript. LC: performed follow-up. CN revised the manuscript.

All authors read and approved the final manuscript.

## Pre-publication history

The pre-publication history for this paper can be accessed here:

http://www.biomedcentral.com/1471-2407/10/324/prepub
